# Estrogen Receptor β in Melanoma: From Molecular Insights to Potential Clinical Utility

**DOI:** 10.3389/fendo.2016.00140

**Published:** 2016-10-26

**Authors:** Monica Marzagalli, Marina Montagnani Marelli, Lavinia Casati, Fabrizio Fontana, Roberta Manuela Moretti, Patrizia Limonta

**Affiliations:** ^1^Department of Pharmacological and Biomolecular Sciences, Università degli Studi di Milano, Milano, Italy; ^2^Department of Medical Biotechnologies and Translational Medicine, Università degli Studi di Milano, Milano, Italy

**Keywords:** melanoma, ERβ, BRAF, NRAS, oncogenic mutations, targeted therapy, ERβ ligands, phytoestrogens

## Abstract

Cutaneous melanoma is an aggressive tumor; its incidence has been reported to increase fast in the past decades. Melanoma is a heterogeneous tumor, with most patients harboring mutations in the BRAF or NRAS oncogenes, leading to the overactivation of the MAPK/ERK and PI3K/Akt pathways. The current therapeutic approaches are based on therapies targeting mutated BRAF and the downstream pathway, and on monoclonal antibodies against the immune checkpoint blockade. However, treatment resistance and side effects are common events of these therapeutic strategies. Increasing evidence supports that melanoma is a hormone-related cancer. Melanoma incidence is higher in males than in females, and females have a significant survival advantage over men. Estrogens exert their effects through estrogen receptors (ERα and ERβ) that affect cancer growth in an opposite way: ERα is associated with a proliferative action and ERβ with an anticancer effect. ERβ is the predominant ER in melanoma, and its expression decreases in melanoma progression, supporting its role as a tumor suppressor. Thus, ERβ is now considered as an effective molecular target for melanoma treatment. 17β-estradiol was reported to inhibit melanoma cells proliferation; however, clinical trials did not provide the expected survival benefits. *In vitro* studies demonstrate that ERβ ligands inhibit the proliferation of melanoma cells harboring the NRAS (but not the BRAF) mutation, suggesting that ERβ activation might impair melanoma development through the inhibition of the PI3K/Akt pathway. These data suggest that ERβ agonists might be considered as an effective treatment strategy, in combination with MAPK inhibitors, for NRAS mutant melanomas. In an era of personalized medicine, pretreatment evaluation of the expression of ER isoforms together with the concurrent oncogenic mutations should be considered before selecting the most appropriate therapeutic intervention. Natural compounds that specifically bind to ERβ have been identified. These phytoestrogens decrease the proliferation of melanoma cells. Importantly, these effects are unrelated to the oncogenic mutations of melanomas, suggesting that, in addition to their ERβ activating function, these compounds might impair melanoma development through additional mechanisms. A better identification of the role of ERβ in melanoma development will help increase the therapeutic options for this aggressive pathology.

## Introduction

Human malignant melanoma is a very aggressive human cancer; its incidence has been found to increase faster than any other cancer during the past decades; importantly, it is one of the most frequent cancers in adolescents and young adults (1). Although it is less common than other malignancies of the skin, melanoma accounts for nearly 75% of skin cancer-related deaths (2).

The incidence rate of cutaneous melanoma is correlated with race; it has been consistently reported that White populations have a greater (10-fold) increase than Hispanian, Black, or Asian populations (3, 4). Based on this observation, the phenotype is considered one of the major risk factors; in particular, fair skin, red or blond hair, blue eyes, and freckles are classic presenting features of melanoma patients (5, 6). In addition to these host factors, the individual risk of developing melanoma also depends on factors such as sun exposure, family history, genetic factors, and their interaction (7).

Epidemiological studies have pointed out the relationship between intense ultraviolet-B (UVB) radiation exposure and melanoma development. In recent years, intense exposure to natural (suntans, sunburns) or artificial (indoor suntannings) UVB have increased in adolescents and young adults, and this seems to be correlated with the increased incidence of melanoma in these population subgroups (8, 9). Family history increases the personal risk of melanoma by three to eight times; this risk increases with the number of affected family members (10). Several genes have been implicated in the development of melanoma: genes involved in the MAPK pathway (*RAS* and *BRAF*, the most frequent mutations), *CDKN2A* [a cyclin-dependent kinase (CDK) gene] genes that are associated with nevi and pigmentation traits, such as *MTAP, MC1R*, and *TYR* (11–14).

## Molecular Aspects of Melanoma Development and Progression

Cutaneous melanoma arises from the malignant transformation of melanocytes, the melanin producing cells of the skin. Melanin (i.e., brown/black eumelanin) is the photoprotective pigment that provides attenuation of UV radiations. In response to UV radiation, keratinocytes secrete factors that regulate melanocytes survival, differentiation, proliferation, and motility. Mutations in genes involved in the processes of melanoma development and progression are very frequently found in melanoma patients. Melanoma is now recognized as a very heterogeneous tumor; however, the majority of patients harbor driver oncogenic mutations at the level of genes encoding for proteins involved in the growth factor receptors signaling pathways (MAPK/ERK and PI3K/Akt) (15–17).

### BRAF Mutations

The MAPK/ERK pathway includes the small G protein RAS and the kinases RAF, MEK, and ERK. BRAF is mutated in approximately 50% of melanomas; 80–90% of these activating mutations involve a single substitution of valine in position 600 with glutamic acid (V600E) (18). Additional, more rare, BRAF mutations include V600K (valine substituted with lysine) and V600D (valine substituted with aspartic acid) (19). BRAF mutations mimic phosphorylation on the regulatory domain of the protein; this leads to an enhanced kinase activity of the protein and activation of its downstream targets MEK and ERK. Activation of this pathway triggers the G1/S transition of the cell cycle through the synthesis of cyclin D1 and negative regulation of the cell cycle inhibitor p27 (20).

### RAS Mutations

RAS was the first oncogene to be identified in melanomas (21). Mutations causing the constitutive activation of this small G protein lead to the hyperactivation of its two downstream pathways, the MAPK/ERK and PI3K pathways, involved in the control of both the proliferative and metastatic behavior of tumor cells. NRAS is the most frequently (about 20–30% of tumors) mutated isoform of the RAS family members in melanoma; very recently, an increase in *NRAS* mutant allele percentage during melanoma progression has been reported (22).

### Other Genetic Mutations

KIT is a receptor with tyrosine kinase activity; it is involved in the development of melanocytes, controlling their proliferation, survival, and migration. It is coupled with the MAPK/ERK, PI3K/Akt, and JAK/STAT intracellular signaling pathways. The KIT receptor is mutated in approximately 15% of mucosal, acral, and chronic sun-damaged melanomas (23). The presence of KIT mutations is particularly interesting because they usually are mutually exclusive with NRAS and BRAF mutations and because of the availability of specific KIT kinase inhibitors in the clinic. Some KIT mutations are well characterized, others are still poorly described (24).

The PI3K/Akt signaling pathway is negatively regulated by PTEN, a tumor suppressor protein. Mutations as well as deletions of PTEN are found in approximately 30% of melanoma cell lines and are frequently associated with mutations in BRAF (24).

The *CDKN2A* is the primary familial high-risk melanoma susceptibility locus identified in families with different cases of melanoma. This gene encodes two suppressor proteins: p16 and p14, involved in the control of cell cycle progression. Specifically, p16 normally inhibits the CDKs, leading to G1/S cell cycle arrest. The second suppressor protein, p14 blocks the degradation of p53, leading to increased apoptosis. In the general population, the prevalence of *CDKN2A* mutations in primary melanomas is only 1.2%; however, germ-line mutations in this locus were reported in approximately 20–57% of families with at least three cases of melanomas (24, 25).

In addition to specific gene mutations, the status of DNA methylation of cutaneous melanoma has been extensively studied and shown to possess both prognostic and therapeutic relevance. Alterations of DNA methylation, histone modifications, and modified expression of microRNAs are well-established epigenetic mechanisms of cell neoplastic transformation. Melanoma cells present aberrant DNA methylation patterns with DNA hypermethylation at the level of CpG islands in the promoter of tumor suppressor genes (leading to their inactivation) and global DNA hypomethylation (contributing to genomic instability). Hypomethylation of specific genes was also reported, leading to the overexpression of normally silenced oncogenes (26, 27). Global DNA hypomethylation was shown to correlate with melanoma progression toward the most aggressive phase and with less favorable clinical outcomes (26, 28).

## Current Therapies for Advanced Melanoma

### Targeting Proliferative Pathways

The majority of melanomas are diagnosed in the early stage (*in situ* melanomas) and are treatable with surgical removal. On the other hand, the prognosis of highly aggressive, late stage melanoma is still poor (29). Prior to 2010, the systemic treatment of choice for advanced metastatic melanoma was limited to cytotoxic chemotherapy and traditional forms of immunotherapy (interleukin-2, IL-2; interferon α-2b, IFN α-2b). Alkylating agents, such as dacarbazine and its prodrug temozolomide, were approved in 1974 by the US FDA; however, the responses to this treatment were very short and less than 5% of patients could achieve a complete response (30, 31). Moreover, chemotherapy treatments were reported to be associated with severe side effects (32, 33) and a very rapid tumor relapse (34). In 1998, the US FDA approved IL-2 for the treatment of metastatic melanoma, based on clinical observations demonstrating sustained remissions in approximately 5–10% of patients (35). However, IL-2 therapy is associated with substantial toxicity, requiring its administration in an intensive care unit setting (36). A combination therapy of dacarbazine with IL-2 or IFN α-2b was reported to improve the progression-free survival but not the overall survival and to be associated with severe side effects (36, 37).

These disappointing results, together with the very quick advances in the dissection of the heterogeneity of melanomas (38) and the understanding of the molecular aspects of melanoma development and progression (24), stimulated the search for newer treatment strategies.

Thus, therapies were developed to specifically target (“targeted therapies”) melanomas harboring either the BRAF or the NRAS mutation (17, 39–42). Vemurafenib, the first targeted drug for melanoma, is a selective BRAF V600E inhibitor, approved by the FDA in August 2011. This drug inhibits the kinase domain of the mutated protein, decreasing cell proliferation through reduced activation of the downstream MAPK/ERK signaling pathway. Encouraging results were reported in phase I clinical trials, showing that vemurafenib was associated with a very good response rate (43); however, later phase trials underlined a short duration of response with a quick development of drug resistance, leading to only marginal patient benefit (44). Dabrafenib is a reversible ATP-competitive inhibitor of V600E- and V600K-mutant BRAF that was approved in 2013; however, the median progression-free survival in melanoma patients treated with dabrafenib was found to be shorter than that reported with vemurafenib (44–46).

It is now well documented that long-term exposure of melanomas to BRAF inhibitors is associated with a rapid development of drug resistance, and this is mainly linked to the rewiring of the MAPK/ERK signaling pathway (44). In this situation, RAS and MEK are elevated while tumor suppressors, such as PTEN, are decreased (47, 48), and BRAF/MEK inhibitor (trametinib and cobimetinib) combinations are now accepted as the standard treatment for resistant melanomas (49). The dabrafenib/trametinib and vemurafenib/cobimetinib combinations were approved by FDA in 2014 and 2015, respectively (17).

As mentioned above, NRAS is mutated in 20–30% of melanoma patients. Unfortunately, so far, the attempts to target mutated NRAS have not led to specific therapeutic strategies; for this reason, the current treatment options for these tumors are mainly focused on targeting the NRAS effector pathways with RAF/MEK and PI3K/Akt inhibitors, either as single agents or as combinations (50). The identification of novel compounds specifically targeting the NRAS-mutant signaling pathway represents a current emerging challenge for the treatment of these melanomas.

### Targeting Immunity Pathways

The lack of the achievement of convincing results with targeted therapies stimulated the search of novel therapeutic approaches, leading to the resurgence of antibody-based immune therapies. Several immune targeted therapies implicate the development of recombinant, humanized, and monoclonal antibodies against specific proteins of the immune cells, such as CTLA-4 and PD-1 (37, 41). The cytotoxic T-lymphocyte antigen 4 (CTLA-4) is an inhibitor checkpoint receptor, expressed on the surface of T cells, that blocks T cell activation and helps regulate the balance between immune activation and tolerance. T cell activation is regulated by signals, provided by CD80 and CD86, located on the surface of antigen-presenting cells (APC), which bind to CD28 on the membrane of T cells. In this setting, T cells are stimulated to enter the cell cycle, differentiate, and produce cytokines (IL-2). In normal conditions, CTLA-4 replaces CD28 in the binding to CD80 and CD86, providing an autocrine regulatory mechanism for preventing uncontrolled T cell activation (51). The anti-CTLA-4 monoclonal antibody, ipilimumab, increases T cell activity and leads to tumor regression (37, 52). In 2011, the FDA approved ipilimumab for the treatment of advanced melanoma, despite its associated side effects related to autoimmune events (colitis) and a poor response rate in patients (53). Similar to CTLA-4, the programed death-1 (PD-1) receptor is a coinhibitory protein that is expressed on the surface of antigen-specific CD8^+^ T cell. The PD-1 ligands (PD-L1 and PD-L2) expressed by tumor cells interact with this receptor, thus triggering inhibitory signals in T cells and resulting in the protection of cancer against immune cell-mediated death (54). Nivolumab and pembrolizumab, two specific monoclonal antibodies against the PD-1 receptor, release the immune checkpoint blockade, thus inducing an anticancer effect (55).

In order to overcome both drug resistance development and side effects, the challenge for basic researchers and clinicians will be the development of novel combination therapies (immune/immune or immune/target specific therapies) for the achievement of both long-term and safe responses (56–58).

Moreover, highlighting additional molecular pathways involved in melanoma growth and progression is urgently foreseen to help increase the development of novel targeted therapeutic strategies for this aggressive pathology.

## The Sex Hormone Milieu and Melanoma

Although melanoma is classically considered a non-hormone-related cancer, growing evidence supports a direct correlation between sex hormones levels (estrogens, in particular) and melanoma growth and progression (59, 60). Epidemiological analyses pointed out a significant divergence in melanoma incidence between sexes in the past three decades. The Surveillance Epidemiology and End Results (SEER) data indicate that, during this period, melanoma incidence was nearly twofold higher in males than in females (61). Gender differences are also observed regarding the age-dependent onset of melanoma, with slightly higher rates in women aged 20–45 that decrease after the age of 45 years. On the other hand, in males, melanoma incidence progressively increases after 45–50 years of age and dramatically increase in men aged 50–85 (61). Moreover, a significant disparity has also been noted in the prognosis of this tumor between males and females, with women having a significant survival advantage over men (62–64). Finally, studies performed in a melanoma fish model (*Xiphophorus couchianus*) showed a twofold lower incidence of melanoma in females than in males after acute exposure to UVB irradiation, and this was accompanied by a sex-specific molecular genetic response (65).

A meta-analysis by Gandini et al. (66) summarizes the evidence pointing out the interaction between endogenous and exogenous estrogens, taking into account natural reproductive factors (menarche, fertility, parity, pregnancy, and menopausal status) and the use of oral contraceptives or hormonal replacement therapy.

Clinical studies documented that melanocytic nevi grow and darken during pregnancy. Melanoma neoplasms are thicker during pregnancy than those diagnosed outside of pregnancy (67), and this is in line with the observation that diethylstilbestrol enhances melanomagenesis in mouse B16 melanoma cells (68). However, it is still unclear whether melanoma patients during pregnancy have worsened outcomes with respect to non-pregnant patients (67, 69, 70). Similar contrasting results were reported on the incidence of melanoma during menopause (71).

A number of studies have focused on the possible link between sex steroid assumption and melanoma development. Most of these studies pointed out that the use of exogenous female hormones (either as oral contraceptives or as hormonal replacement) do not contribute to increased risk of cutaneous melanoma (66, 72). Finally, Auriemma et al. (73) recently investigated the possible mole modifications in women undergoing controlled ovarian stimulation (COS) for assisted reproduction technologies. The conclusion raised by these authors is that the results obtained do not support a causal relation between the supraphysiological hormone levels stimulation and worsening of clinical features of moles.

Another issue that needs to be solved is the expression/activity of aromatase in melanoma tissues. It seems that the skin has its own capacity to produce steroids (including estrogens) (59). In line with this, Santen and coworkers reported that the aromatase enzyme is expressed in melanoma tissues (74); however, no correlation was found in this paper between the expression of this enzyme and clinical outcomes. It must be underlined that this study did not have any follow-up; moreover, the first generation aromatase inhibitor aminoglutethimide was found to be ineffective in reducing melanoma progression (75). Further studies performed with newer aromatase inhibitors will likely help clarify this issue.

In conclusion, although contrasting results were so far reported on the relationship between estrogen levels and melanoma risk, the influence of the endocrine status on the development of melanoma is now well accepted. Based on these observations, de Giorgi and coworkers suggested that cutaneous melanoma should be considered as a hormone-related tumor (76), although this conclusion is mainly supported by the results from the studies analyzing the estrogen receptor (ER), particularly the ERβ, status of melanomas.

## Estrogen Receptors α and β: Opposite Roles in Cancer

Estrogens exert their biological effects through two ER, ERα and ERβ, members of the nuclear receptor superfamily of transcription factors (77). ERα was cloned in 1985, and it was considered the only receptor responsible for estrogens action (78). ERβ was cloned from a rat prostate cDNA library in 1996, and this opened the way to the discovery of the human counterpart (79, 80). Both ERs are nuclear receptors, which can form either homo- or heterodimers and, upon activation, translocate into the nucleus to bind with coregulatory proteins and control the transcription of target genes through the binding to specific ERE regions (81–84).

The two receptors are encoded by two different genes (*ESR1* and *ESR2*) that are located on chromosomes 6 and 14, respectively. They share the same general structure, characterized by three (independent, but interacting) functional domains: the N-terminal domain (NTD or A/B) containing a transactivation domain and a domain responsible for the recruitment of coactivators/corepressors; the DNA-binding domain (DBD or C) containing zinc fingers, necessary for the binding of the receptors to the estrogen response elements in the promoter region of target genes; the ligand-binding domain (LBD or D/E/F) with a ligand-dependent transactivating function. This LBD domain is also responsible for the binding to co-regulatory and chaperone proteins (77). The two ERs share about 97% similarity in the DBD, 59% in LBD, and only 16% in their NTD (77, 85). The differences in the LBD are responsible for the shape of the ligand-binding pocket and, based on this observation, specific ligands for each receptor subtype have been designed and synthesized (86, 87). At the same time, the same ligand may have different binding affinity for ERα or ERβ subtypes.

It has been widely reported that the endogenous hormone estradiol binds to both receptors with a similar binding affinity and the same transactivational activity in different cell types (88, 89). Thus, the specific effects of endogenous estradiol on ERα and ERβ activation largely depend on the different cell contexts and specifically on the recruitment of cell type-specific coactivators/corepressors as well as chromatin remodeling proteins (90). In melanoma cells, the binding affinity of estradiol for ERα and ERβ has not been specifically evaluated, but it is expected to be the same as in other cell types. However, it must be underlined that the majority of the studies so far performed pointed out a prevalence of expression of ERβ in these cells (see below).

Synthetic or natural ligands bind to ERα or ERβ with different affinities according to their chemical structure (88). Antiestrogens, such as tamoxifen, raloxifene, and ICI-164,384 are partial agonists/antagonists of ERα, depending on the target tissue, and are referred to as selective estrogen receptor modulators (SERMs). Different natural compounds [genistein, apigenin, liquiritigenin (LQ), silibinin, and silymarin] were reported to bind with higher affinity and, therefore, to specifically activate the ERβ subtype; for this reason, they are referred to as estrogen receptor subtype agonists (ERSAs) (86, 91, 92). Thus, in a given cell, the different binding affinity and transactivational activity of synthetic or natural ligands on the two ER subtypes seem to depend on the ERα/ERβ ratio as well as on the specific cell context (88, 90, 93). The full-length ERβ protein, also named ERβ1, is generated from 8 exons and includes 530 aminoacids. After ligand binding, ERβ1 can form either homodimers or ERα/ERβ1 heterodimers, which bind to ERE sequences on DNA; such interactions contribute in modulating ERβ1/ERα activity, in a specific cell context (94). In addition to ERβ1, four alternative splice variants of ERβ (ERβ2, ERβ3, ERβ4, and ERβ5) have been identified (95–97). All these variants have a truncated C-terminus and, for this reason, no ligands have been found for these forms; moreover, they are unable to form homodimers (97, 98). However, ERβ2 and ERβ5 can form heterodimers with ERβ1 isoform or with ERα, thus inhibiting their binding to ERE elements on DNA (96, 97). ERβ5 was reported to have a stable expression during the process of carcinogenesis, in contrast to ERβ1 and ERβ2 (99).

**Figure 1:**
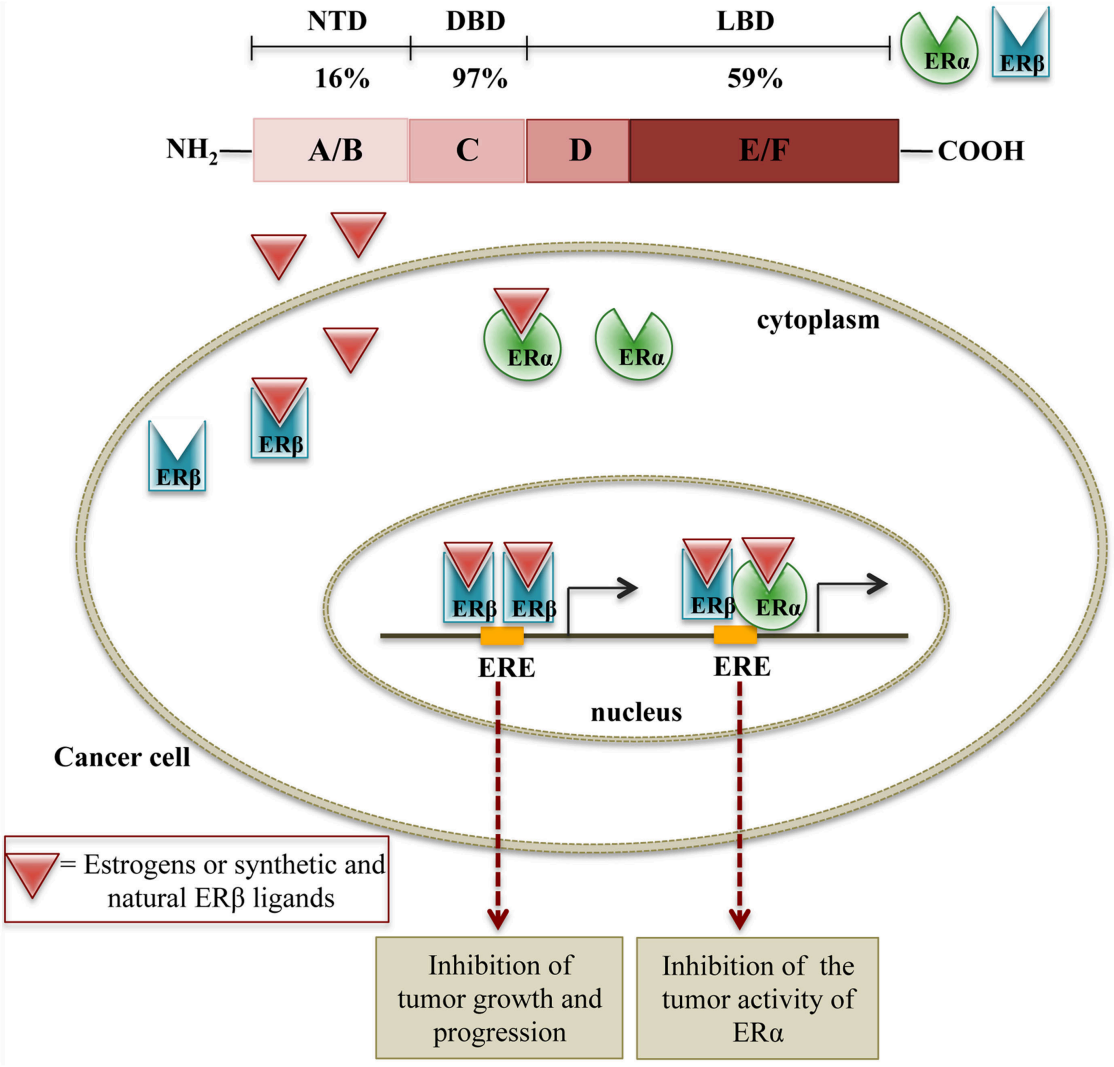
**Schematic representation of the molecular mechanisms of ERβ activation on cell growth**. In cancer cells expressing both ERβ and ERα, ERβ ligands can either induce the formation of ERβ:ERβ homodimers, inhibiting cell proliferation or ERβ:ERα heterodimers, counteracting the proliferative activity of ERα.

It is now clear that ERα and ERβ are associated with different activities, according to their specific tissue distribution (77, 94, 100). Increasing evidence supports a relationship between the perturbation of estrogen signaling and cancer initiation, promotion, and progression. Specifically, the variation of the ratio ERα/ERβ in tumor tissues supports the notion that the two ER subtypes have different functions in cancer biology and therapy. Overall, it is now well accepted that ERα contributes to tumorigenesis by stimulating cell proliferation, while ERβ is endowed with a significant antitumor activity. Thus, both synthetic and natural ERβ ligands may interfere with the mechanisms of tumor growth either by activating ERβ or by interfering with the tumor activity of ERα through ERα/ERβ heterodimers formation (77, 94, 101) (Figure 1).

## Estrogen Receptor β and Melanoma

ERα is the main ER in human skin; however, this receptor does not seem to play any role in the pathophysiology of melanoma precursor lesions or melanomas. On the other hand, ERβ has been reported to be the predominant ER subtype in melanocytic lesions; its distribution and levels of expression are different in the different classifications of the lesions. In 2006, Schmidt and coworkers investigated the expression of ERα and ERβ in benign nevi, dysplastic nevi with mild/moderate/severe cytological atypia, lentigo malignas, and melanomas with different depth (Clark) and thickness (Breslow) (102). They found that ERβ, but not ERα, was the predominant ER in both benign and malignant lesions and that ERβ expression levels also correlated with the tumor microenvironment. In line with these observations, it was reported that melanocytic nevi and malignant melanomas are both positive for ERβ, while they are negative for ERα (103). de Giorgi and coworkers investigated the expression of ERα and ERβ in human melanoma tissues (104). They found that both receptors are expressed at the mRNA level, but only the ERβ protein was present in these tissues. Analyzing melanoma cases into two groups according to Breslow thickness (104), these authors observed that the levels of both ERβ mRNA and protein were lower in thicker and more invasive tumors. According to these authors, these data support a protective role for ERβ in the metastatic process of melanoma cells (104, 105). An opposite correlation between ERβ levels and Breslow thickness was also reported by Schmidt et al. (102). Moreover, ERβ expression was found to be lower in tumor tissues compared with the adjacent healthy skin. Men showed lower levels of ERβ than women in both melanoma and healthy tissues, in agreement with sex differences in melanoma survival (106). ERβ has been found to be expressed in melanomas of pregnant women more frequently than in men and also a trend to a higher expression in women than in men has been reported. This indicates that ERβ might explain the generally favorable prognosis of melanoma in women (107). ERβ expression has been recently shown to be downregulated in aggressive, metastatic melanomas, suggesting its possible utility as a marker for metastatic potential and for prognosis in malignant melanomas (108). Finally, a polymorphism at the *Alu*I restriction site was identified in a high proportion of melanoma, suggesting that the polymorphism of this receptor could be related to a higher susceptibility to the development of this tumor (109).

In line with these clinical observations, we recently demonstrated that ERβ, but not ERα, is the ER expressed in human melanoma cell lines, harboring different genetic mutations (A375, BLM, WM115, and WM1552) (110). Moreover, the pattern of expression of the different ERβ isoforms was similar in BLM (NRAS-mutant, BRAF-wild type) and WM115 (BRAF V600D-mutant) melanoma cells: ERβ1 and ERβ5 were found to be expressed at similar levels while ERβ2 showed a higher level of expression. On the other hand, in A375 (BRAF V600E-mutant) cells, both ERβ2 and ERβ5 were expressed at higher levels than ERβ1 (110).

Taken together, these data indicate that ERβ is expressed in melanoma cells, and its levels of expression negatively correlate with melanoma growth and progression, further supporting the notion that this receptor might be endowed with an antitumor activity in melanoma. Similar data have been previously reported for different types of tumors, both related and unrelated to the reproductive system (111–119).

## Antitumor Activity of ERβ in Melanoma

Despite the extensive clinical observations reporting the expression of ERβ in melanoma tissues and its negative correlation with tumor progression, the data so far available on the direct antitumor effects of ERβ ligands are still scanty.

As mentioned above, both genomic and non-genomic signaling pathways mediate the effects of ERs activation. The genomic effects of ERs activation are mediated by receptor dimerization and binding to ERE on DNA while interacting with transcriptional coactivators, to regulate gene transcription. The non-genomic effects of ER ligands are mediated by two main signaling pathways: RAS/RAF/MEK/ERK and PI3K/Akt. Specifically, the PI3K/Akt pathway is well known to mediate the antitumor activity of ERβ in several types of cancers (94, 120). As discussed, some of the proteins involved in these signaling cascades are mutated in the majority of melanoma. Thus, both the ERs and the MAPK and PI3K pathways might represent molecular targets to counteract melanoma growth and progression.

Although extensive epidemiological studies clearly indicate that ERβ is the main ER subtype to be expressed in melanoma, the data so far available on the possible effect of estrogens on the growth and progression of this tumor are still scanty. Sarti and coworkers reported that 17β-estradiol exerts a significant inhibitory activity on the proliferation of the human SK-Mel 23 melanoma cell line, expressing ERβ, but not ERα (referred to as “type II estrogen binding site” by these authors) (121). The sex hormone 17β-estradiol was also shown to reduce the invasive behavior of human melanoma cells lacking the ERα receptor (122). *In vitro* and *in vivo* antitumor effects on melanoma were also reported for 2-methoxyestradiol, an endogenous metabolite of estradiol; however, it must be pointed out that the antitumor activity of this compound was not found to be mediated by ERs (both α and β) activation (123, 124).

The ER antagonist tamoxifen was first shown to induce cell death in human malignant melanoma cells, possibly through inactivation of the IGF-I receptor (125). This antiestrogen was also reported to reduce tumor cell metastatic behavior in the mouse melanoma cell line B16BL6 (126). However, later clinical studies did not confirm these promising results from *in vitro* studies. The effects of chemotherapy with and without tamoxifen for the treatment of aggressive melanoma were compared in different clinical trials. These studies reported that co-treatment with tamoxifen may provide improvements in response rates, although it is often accompanied by increased toxicity and no survival benefit (60, 127–131). It has been proposed that this is due to the fact that tamoxifen may decrease cell proliferation when it binds to ERα while it may increase cell proliferation when it binds to ERβ (60). Thus, the antitumor vs. prosurvival effects of tamoxifen likely depend on the different ERα/ERβ ratios in a given tissue (105). In line with this observation, low levels of expression of ERβ were shown to correlate with tamoxifen resistance in breast cancer cells (132, 133).

**Figure 2:**
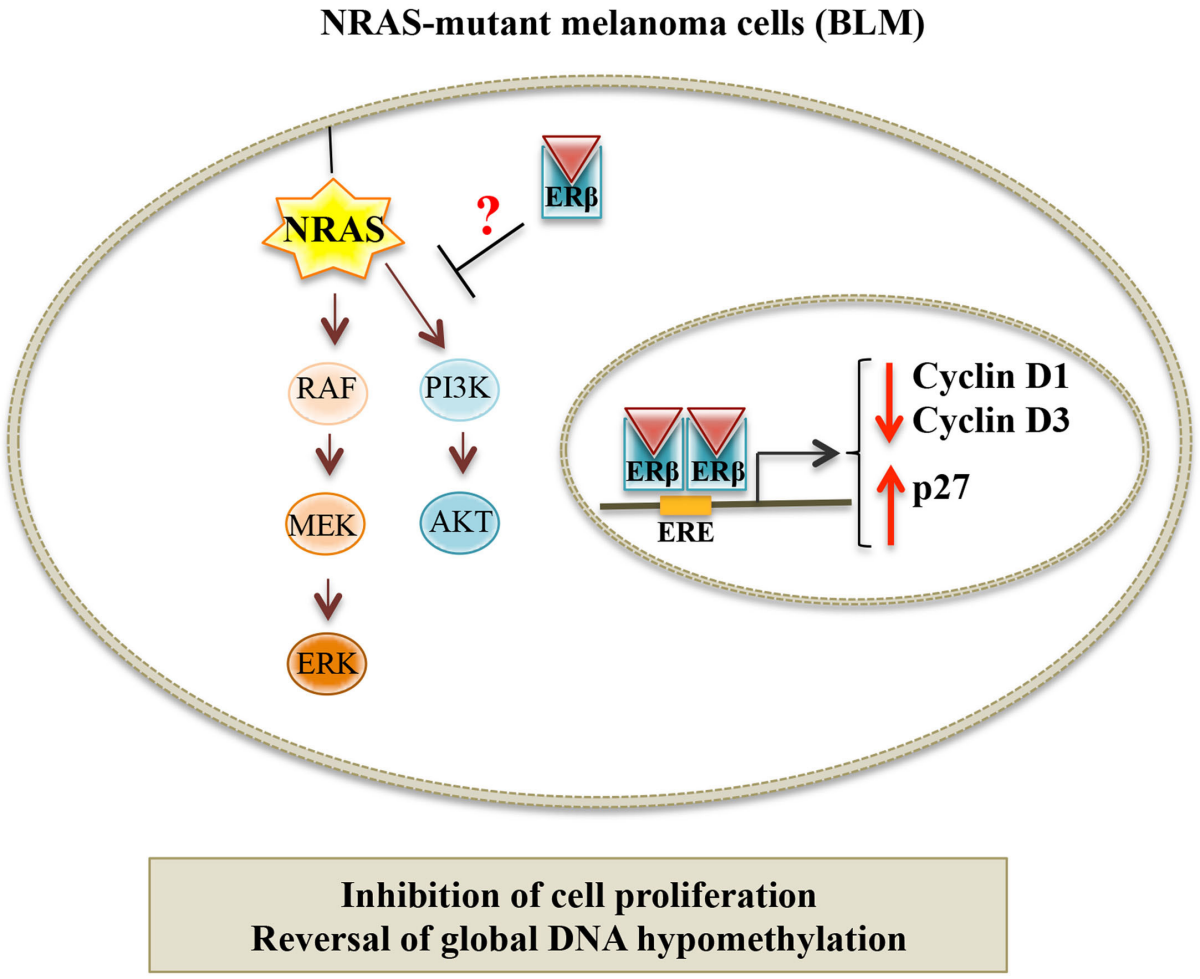
**Proposed model for the targeting of NRAS-mutant melanoma by ERβ ligands**. In NRAS-mutant melanoma cells (BLM), ERβ agonists trigger genomic effects at the nuclear level by modulating the expression of cell cycle-related proteins and by reversing the global hypomethylation status of these cells. Moreover, it is hypothesized that activated ERβ might also exert non-genomic effects, by interfering with the PI3K/Akt signaling pathway, as previously described for different cancer cell lines expressing this receptor.

In a recent paper, we showed that ERβ, but not ERα, is the ER expressed in human melanoma cell lines, harboring different genetic mutations. We demonstrated that, in BLM (BRAF-wild type, NRAS-mutant) melanoma cells, ERβ agonists [17β-estradiol, diarylpropionitrile (DPN), KB1] significantly inhibited cell proliferation, and this effect was abrogated by an ER antagonist. ERβ activation triggered the translocation of the receptor from the cytoplasmic to the nuclear compartment and its transcriptional activity. The activity of ERβ agonists was accompanied by an altered expression of the proteins involved in the G1/S transition of the cell cycle (decreased levels of cyclin D1 and cyclin D3, and increased expression of p27, a CDK inhibitor); the apoptosis pathway was not involved in this activity (no change in the cleaved, active form of caspase-3). Importantly, we reported an aberrant global DNA hypomethylation in BLM cells, indicative of genome instability; ERβ activation reverted this hypomethylation status (110). Taken together, these data demonstrate that, in NRAS-mutant melanoma cells, ERβ is associated with an antitumor activity, by causing cell cycle arrest and through the regulation of cell cycle-associated proteins; similar observations were previously reported in different types of cancers expressing this receptor (94, 101, 134–140). Surprisingly, in our paper, we could also show that ERβ agonists were ineffective in reducing the proliferation of A375 and WM1552 (V600E BRAF-mutant) melanoma cells, expressing the ER isoform. We speculated that the different effects of ERβ ligands in these cell lines might be related to their specific oncogenic mutation status. Actually, as mentioned above, NRAS and BRAF mutations are the most frequent oncogenic mutations found in melanoma. NRAS mutations have been reported to be associated with increased activation of the two main downstream pathways: PI3K/Akt and MEK/ERK. On the other hand, in melanoma cells harboring BRAF mutations, only the MEK/ERK signaling cascade was shown to be overactivated. In agreement with our data, ERβ ligands have been previously shown to exert their antitumor effects through inactivation of RAS as well as of the PI3K/Akt pathway in some cancer cells (94, 141, 142). Moreover, Wang and coworkers (143) have recently reported an inverse correlation between the expression of ERβ and the activity of the PI3K/Akt pathway in aggressive, triple-negative, breast cancer. Chen et al. (141) demonstrated that, in breast cancer cells, the isoflavone calycosin activates ERβ, and this is followed by a decreased activity of the PI3K/Akt pathway; on the other hand, calycosin did not affect the activity of the MAPK/ERK cascade. Based on these observations, it seems possible to conclude that ERβ activation might significantly reduce the growth of cutaneous melanoma cells harboring the NRAS mutation, possibly through the inhibition of the PI3K/Akt signaling pathway (Figure 2). On the other hand, ERβ agonists will not be able to reduce the proliferation of melanoma cells carrying the BRAF (V600E) mutation, which is associated with the overactivation of the MEK/ERK signaling cascade.

These data suggest that, in melanoma patients harboring the NRAS mutation, ERβ might represent an effective molecular target for personalized therapeutic interventions. For instance, these interventions might be based on an ERβ agonist given either alone or in combination with specific inhibitors of the MEK cascade (i.e., trametinib and cobimetinib), with the aim to block the activity of both the PI3K/Akt and the MEK/ERK signaling pathways. In an era of personalized medicine, it is suggested that a pretreatment evaluation of the ER isoforms expression in each melanoma patient, together with the concurrent oncogenic mutations, should be considered in order to anticipate the response of melanoma patients to novel therapeutic strategies (50).

## Natural ERβ Ligands and Melanoma

Phytoestrogens are natural, plant-derived, estrogenic compounds that preferentially bind to ERβ than ERα (91, 144). These compounds have been suggested as possible effective agents in the prevention of several diseases, such as menopausal symptoms, osteoporosis, and cardiac diseases (145). They have also been reported to exert antitumor activity on different types of cancers, based on their high affinity binding to ERβ and their ability to increase the expression of this receptor subtype (115, 119, 138, 146–148) (Table 1).

**Table 1:** **Natural ERβ ligands and melanoma**.

Compound	Cellular model	Effects	Reference
Genistein	Human melanoma cells	Suppression of the growth and the metastatic potential	(158–162, 164, 165)
	Murine B16 melanoma xenografts	Decrease of tumor-associated angiogenesis	
Liquiritigenin	Murine B16-F10 melanoma cells and xenografts	Enhancement of antiproliferative and antimetastatic effects of chemotherapy	(142, 166–168)
Apigenin	Human A375 melanoma cells	Proapoptotic and antimetastatic effects	(155, 179–187)
	Murine B16-BL6 melanoma cells		
Silymarin	Human A375-S2 melanoma cells	Chemopreventive, antitumor, and antimetastatic activity	(200–203)

Thus, the antitumor activity of phytoestrogens largely depends on the subtype of ER expressed in a given tissue, as well as its levels of expression and the circulating steroid hormones milieu (149, 150). As discussed for estrogens, phytoestrogens can exert their effects through both genomic and non-genomic (by affecting different intracellular signaling pathways) activities (151). These effects include alterations of tyrosine kinase pathways, antioxidant effects, and epigenetic mechanisms, and also through recruitment of coregulators endowed with chromatin binding activities, thus underlying the relevance of epigenetic mechanisms in estrogen signaling (152).

Flavonoids are a large subclass of polyphenolic compounds present in many vegetables and medicinal herbs (153); some of these compounds have been demonstrated to play a potential role in the management of different types of tumors with little side effects since they interfere with cancer progression by regulating cell proliferation, apoptosis, invasion, and metastasis (154, 155).

The phytoestrogens *genistein* and *daidzein* are isoflavones, found in soybean, one of the most important food components in Asian diet (156, 157). These compounds received considerable attention based on epidemiological studies demonstrating that the soybean-containing diets were associated with a lower incidence of particular cancers in Asian population (158). Genistein interacts with both ERα and ERβ but has higher affinity for ERβ (138, 159).

This isoflavone was first shown to suppress the growth and also the metastatic potential of human melanoma cells, both *in vitro* and *in vivo*, with or without the induction of differentiation, dendritic formation, etc. (160–162).

It is well known that phytoestrogens undergo glycosidic binding to carbohydrates to form molecules that cannot be easily absorbed. For this reason, the glycosidic binding must be broken up by enzymes (glycosidases) present in the gut, produced by the intestinal microflora, that transform the glycosidic forms in the corresponding “aglicones” that are very easily absorbed (163). However, it has also been reported that genistin, as other flavonoid glycosides, is partly absorbed without previous cleavage and does not need to be hydrolized to be biologically active. In line with these observations, Russo and coworkers (158) reported that genistin exerts an inhibitory effect of human melanoma cells and inhibited ultraviolet (UV) light-induced oxidative DNA damage. Moreover, genistin and daidzin (the glycosidic forms of the two isoflavones) showed a protective effect on DNA damage; only genistin, but not daidzin, was able to counteract the proliferation of human melanoma cells (M14). These data suggest that not only the isoflavone aglycons but also the corresponding glycosides may trigger significant antitumor effects in melanoma cells. Genistein, at non-cytotoxic concentrations, was reported to reduce the growth as well as the motility of mouse B16 melanoma cells, through modulation of the activity of enzymes involved in the degradation of the extracellular matrix [i.e., urokinase-type plasminogen activator (uPA)]. Moreover, *in vivo* i.p. administration of genistein was found to decrease tumor-associated angiogenesis in nude mice-bearing mouse melanoma xenografts (B16); similar results were obtained with a soybean-based diet (164, 165).

The flavanone LQ is extracted from conventional herbal medicine-glycyrrhizae and is considered a highly selective ERβ agonist (166). In a recent paper, Shi and coworkers (167) investigated whether LQ may potentiate the antimetastatic effect of chemotherapeutic drugs [cis-diamine dichloroplatinum (CDDP)] in murine B16F10 melanoma cells. It was found that LQ significantly potentiates the anti-migratory/anti-invasive effects of CDDP, and this is mediated by downregulation of the PI3K/Akt pathway. LQ also enhanced the CDDP-induced suppression of lung metastasis formation in nude mice bearing B16F10 xenografts.

It has been reported that LQ displays only 20% greater binding affinity for ERβ than ERα (166); however, recent data demonstrate that different molecular mechanisms, in addition to its ability to bind ERβ, can account for its activity. In particular, it has been recently shown that the LQ/ERβ complex is able to recruit ERβ coactivators and that it specifically binds to ERβ responsive elements in the promoter region of target genes (159, 166, 168).

As a further support to the notion that ERβ is involved in LQ antitumor activity, data from the literature demonstrate that, in temozolomide-resistant U138 glioma cells, LQ treatment sensitizes cancer cells to drug-induced inhibition of cell proliferation and significantly increases ERβ expression. Moreover, LQ treatment significantly inhibits the activity of the PI3K/Akt pathway, and this effect is completely reversed by ERβ knockdown (142).

On the other hand, LQ is known to have a lower affinity for ERβ than estradiol (169); this raises the question of the dose of this compound that should be necessary to exert a physiological/therapeutic effect. Although further studies are required to specifically answer this question, it must be underlined that LQ has been reported to inhibit tumor growth in preclinical mouse models of gliomas and human cervical cancers (138, 170, 171).

Liquiritigenin is a component of licorice roots, and *licorice root extracts* are utilized by menopausal women as dietary supplements to fight menopausal symptoms as an alternative to classical pharmacological interventions (172, 173). In addition to LQ and iso-liquiritigenin, licorice extracts contain a series of different compounds, such as glycyrrhizin, glabridin, glabrene, calycosin, methoxychalcone, vestitol, and glycycoumarin. Glycyrrhizin, when consumed at high doses and for a long period, is associated with serious side effects, such as hypertension. Therefore, attention has been recently given to the other components of licorice root extracts with the aim to investigate their possible interaction with the ER system. Most of these compounds have been reported to behave as SERMs, specifically acting as ERα agonists in some cell types while behaving as ERα antagonists in other cell contexts (169, 174, 175).

Whether the components of licorice root extracts might exert antiproliferative effects on cancer cells, and particularly on melanoma cells, by interfering with the estrogenic system is still unclear. To this purpose, it must be recalled that ERβ, and not ERα, is the predominant ER subtype in melanocytic lesions (see above). The novel flavonoid isoangustone A has been shown to inhibit the proliferation of human melanoma cells through blockade of cell-cycle progression and downregulation of the PI3K/Akt pathway (176); glycyrrhizin seems to protect human melanoma cells from UVB irradiations (177); more recently, the licorice component licochalcone A has been reported to reduce the growth of different cancer cell lines, including melanoma cells (178). However, the possible involvement of ERβ in the antitumor activity of these compounds still needs to be investigated.

The flavone *apigenin* is another phytoestrogen that received growing attention as a potential chemopreventive agent and suppressor of cancer growth (155, 179–182). In A375 human melanoma cells, apigenin was reported to induce apoptosis through accumulation of reactive oxygen species in mitochondria, upregulation of Bax, caspase 3, 9, and PARP and downregulation of Bcl-2, thus triggering the mitochondrial apoptotic pathway (183). Apigenin was also shown to reduce the metastatic potential of melanoma cells *in vitro* (184) as well as *in vivo* in a preclinical model of mice injected with B16-BL6 murine melanoma cells (185). In addition, the antimetastatic activity of this compound in murine B16F10 melanoma cells has been demonstrated to be mediated by suppression of signal transducer and activator of transcription 3 (STAT3) phosphorylation and downregulation of STAT3 target genes MMP-2, MMP-9, VEGF, and Twist1, known to be involved in the metastatic behavior of cancer cells (186). Moreover, apigenin was found to overcome resistance to the anticancer agent TRAIL (tumor necrosis factor-related apoptosis-inducing ligand), a very promising compound that kills different tumor cells while sparing normal tissues, but is very often associated with development of resistance. Apigenin inhibits the expression of the p53 antagonist murine double minute 2 (Mdm2), thus increasing p53 levels and, consequently, upregulating the TRAIL receptor 2, the p53 target gene (187). However, whether ERβ might be involved in the antitumor activity of apigenin in melanoma is still unclear. On the other hand, this phytoestrogen has been reported to exert its antitumor activity in breast and prostate cancer cells through activation of ERβ (180); interestingly, in prostate cancer cells, apigenin induces apoptosis by selectively inhibiting proteasomal activity, thus rescuing ERβ from degradation and, therefore, increasing its intracellular levels (188).

Apigenin has also been shown to cross-react with progesterone receptors (189). This compound induces apoptosis and blocks the medroxyprogesterone acetate-dependent growth of aggressive breast cancer xenograft tumors (190–193). On the other hand, the expression/activity of the progesterone receptor and the possible cross-reaction of apigenin with this receptor in melanoma cells still need to be clarified. Progesterone receptors have been shown to be expressed in both conjunctival nevi and melanoma specimens. However, the rate of progesterone receptors was not found to correlate with the disease course; progesterone has been reported to inhibit the proliferation of melanoma cells, but this effect was found not to be mediated by the progesterone receptor (194, 195). *Silymarin*, extracted from *Silybum marianum*, is a mixture of four flavolignans (silybinin, isosilybinin, silydianin, and silychristin) and the isoflavone taxifolin. Since the first demonstration that silymarin selectively binds to ERβ (but not to ERα), this mixture was considered an ERβ specific ligand (196). The effects of silymarin were analyzed on ultraviolet light (UV)-induced cell apoptosis in human A375-S2 melanoma cells. It was found that this flavonoid prevents UV irradiation-induced apoptosis of these cells, through the activation of the Akt and SIRT1 pathways (197–199). On the other hand, silymarin has been recently shown to inihibit melanoma cell growth both *in vitro* and *in vivo* (200, 201) and to induce cell cycle arrest in melanoma cells directly targeting the MEK1/2 pathway (202). Moreover, Vaid and coworkers (203) investigated the effects of silymarin on the metastatic properties of human melanoma cell lines. They reported that this polyphenolic flavonoid significantly inhibits the migratory/invasive behavior of these cells, and this antimetastatic behavior is mediated by the Wnt/β-catenin pathway. Taken together, these observations strongly support the notion that the natural ERβ agonist silymarin may exert both a chemopreventive and an antitumor activity in melanoma cells. Similar observations were previously reported for experimental models of colon carcinogenesis (204).

It must be underlined that silymarin can also bind the androgen receptor, and it activates the same molecular pathways involved in the ERβ signaling, specifically in prostate cancer cells (205, 206). However, the expression and the possible role of this receptor in skin carcinogenesis is still a matter of debate (60, 107, 207, 208). Moreover, to the authors’ knowledge, no data are so far available in the literature on the possible role of androgen receptors in the antitumor activity of silymarin in melanoma.

Given the antiproliferative/proapoptotic activity of phytoestrogens in melanoma, as well as in different types of tumors, and based on their high-binding affinity to the ERβ subtype, further studies are needed to definitely confirm the role of ERβ in the antitumor activity of these natural compounds. Results from these studies will likely open the way to novel, nutraceutical-based, chemopreventive and therapeutic (i.e., combinatorial) options for this aggressive pathology.

## Conclusion

Increasing evidence supports a close relationship between the sex hormone (i.e., estrogens and ERs) milieu and melanoma growth and progression. Clinical studies have pointed out that ERβ, but not ERα, is the predominant ER subtype in melanoma tissues, and its levels of expression are downregulated during melanoma progression toward the most aggressive phases. Considering that ERβ is the ER subtype widely shown to be associated with an anticancer effect in tumors in which it is expressed, these clinical observations strongly support the notion that ERβ might be considered as a possible molecular target for the development of therapeutic strategies for melanoma. However, the data so far available on the direct antitumor effects of ERβ ligands in melanoma are still scanty. Clinical studies reported that the ER antagonist tamoxifen provides only variable improvements in the rates of response of melanoma patients to chemotherapy. *In vitro* studies demonstrate that ERβ agonists can impair melanoma cell proliferation, but this depends on the genetic mutational status (NRAS vs. BRAF) of melanoma cells. Taken together, these data strongly support the notion that evaluation of the oncogenic mutation (BRAF vs. NRAS) together with the expression of the ER subtype (ERβ vs. ERα) in melanoma patients should be taken into consideration when considering the most specific therapeutic approach to be applied. Moreover, based on the promising experimental and preclinical data so far available, we believe that a better identification of the molecular mechanisms of the antitumor activity of natural ERβ ligands will likely improve the treatment strategies for melanoma patients.

## Author Contributions

All authors listed have made substantial, direct, and intellectual contribution to the work and approved it for publication. MMM and MM evaluated the paper and designed the figures. PL wrote the paper.

## Conflict of Interest Statement

The authors declare that the research was conducted in the absence of any commercial or financial relationships that could be constructed as a potential conflict of interest. The reviewer JC and handling editor declared their shared affiliation, and the handling editor states that the process nevertheless met the standards of a fair and objective review.
